# Parkin-mediated mitophagy is a potential treatment for oxaliplatin-induced peripheral neuropathy

**DOI:** 10.1152/ajpcell.00276.2023

**Published:** 2023-12-11

**Authors:** Guoqing Zhao, Te Zhang, Jiannan Li, Longyun Li, Peng Chen, Chunlu Zhang, Kai Li, Cancan Cui

**Affiliations:** ^1^Anesthesiology Department, China-Japan Union Hospital of Jilin University, Changchun, People’s Republic of China; ^2^Department of Plastic and Reconstructive Microsurgery, China-Japan Union Hospital of Jilin University, Changchun, People’s Republic of China; ^3^Radiology Department, China-Japan Union Hospital of Jilin University, Changchun, People’s Republic of China

**Keywords:** mitophagy, oxaliplatin, peripheral nerve pain, Parkin, salidroside

## Abstract

Oxaliplatin-induced peripheral nerve pain (OIPNP) is a common chemotherapy-related complication, but the mechanism is complex. Mitochondria are vital for cellular homeostasis and regulating oxidative stress. Parkin-mediated mitophagy is a cellular process that removes damaged mitochondria, exhibiting a protective effect in various diseases; however, its role in OIPNP remains unclear. In this study, we found that Parkin-mediated mitophagy was decreased, and reactive oxygen species (ROS) was upregulated in OIPNP rat dorsal root ganglion (DRG) in vivo and in PC12 cells stimulated with oxaliplatin (OXA) in vitro. Overexpression of Parkin indicated that OXA might cause mitochondrial and cell damage by inhibiting mitophagy. We also showed that salidroside (SAL) upregulated Parkin-mediated mitophagy to eliminate damaged mitochondria and promote PC12 cell survival. Knockdown of Parkin indicated that mitophagy is crucial for apoptosis and mitochondrial homeostasis in PC12 cells. In vivo study also demonstrated that SAL enhances Parkin-mediated mitophagy in the DRG and alleviates peripheral nerve injury and pain. These results suggest that Parkin-mediated mitophagy is involved in the pathogenesis of OIPNP and may be a potential therapeutic target for OIPNP.

**NEW & NOTEWORTHY** This article discusses the effects and mechanisms of Parkin-mediated mitophagy in oxaliplatin-induced peripheral nerve pain (OIPNP) from both in vivo and in vitro. We believe that our study makes a significant contribution to the literature because OIPNP has always been the focus of clinical medicine, and mitochondrial quality regulation mechanisms especially Parkin-mediated mitophagy, have been deeply studied in recent years. We use a variety of molecular biological techniques and animal experiments to support our argument.

## Graphical abstract

**Figure:**
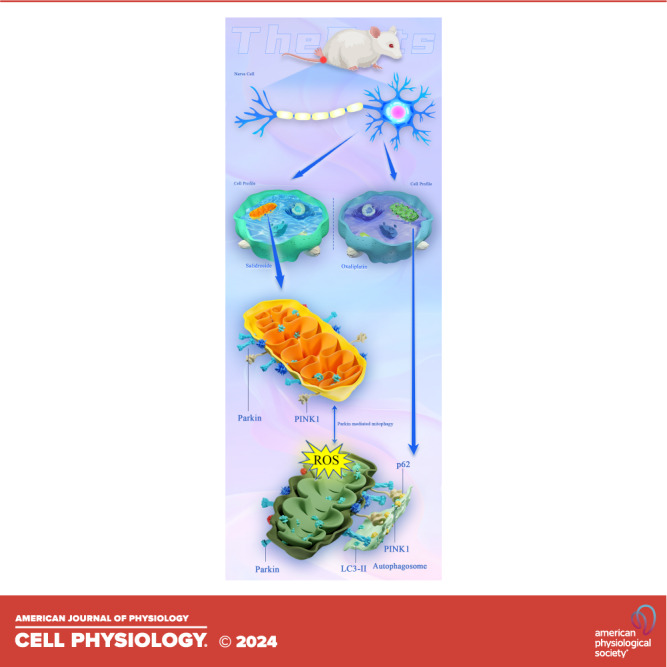

## INTRODUCTION

Chemotherapy-induced peripheral neuropathic pain (CIPNP) is a dose-dependent complication induced by various chemotherapeutic agents (1). Platinum agents are the most neurotoxic, with oxaliplatin (OXA) causing the highest prevalence of CIPNP (2–4). An accumulation of 540–850 mg/m^2^ OXA in the body can develop chronic neuropathy with a sock-glove-like distribution and pain (5). Neuropathy can progressively worsen or lead to permanent neuropathic pain upon drug withdrawal (5). Oxaliplatin-induced peripheral nerve pain (OIPNP) is not only the main reason for drug discontinuation in cancer patients but also severely affects the prognosis and mental health of patients (6, 7). However, the mechanism of OIPNP remains unclear.

Mitochondria are essential subcellular organelles that regulate reactive oxygen species (ROS) production and apoptotic pathways and are important in promoting healthy neuronal growth and neuropathic pain development (8, 9). Previous studies have shown that nerve injury, ion channel changes, and inflammation are closely related to OIPNP (5, 7, 10, 11). In recent years, it has been reported that OXA can form adducts with mitochondrial DNA (mtDNA) in the dorsal root ganglion (DRG), resulting in mitochondrial damage (12, 13). Oxidative stress caused by mitochondrial dysfunction is another important cause of OIPNP (14).

Autophagy is an adaptive mechanism in stressed and damaged neurons (15, 16). Upregulated autophagic activity may help reduce pain behaviors. Mitophagy is the selective phagocytosis of damaged mitochondria, the most classic of which is Parkin-mediated mitophagy (17–19). Zhang et al. suggested that enhancing Parkin-mediated mitophagy could reduce damaged mitochondria caused by cisplatin on PC12 cells and reverse cellular damage (20). However, the changes induced by the Parkin-mediated mitophagy in OIPNP are unknown.

Salidroside (SAL) is a biologically active ingredient extracted from *Rhodiola rosea*, which has anti-tumor, antioxidant, anti-aging, and other pharmacological properties (21, 22). SAL may provide neuroprotection by enhancing Parkin-mediated mitophagy (23, 24). Here, we found a reduced Parkin expression in the DRG of OIPNP rats. Oxaliplatin-induced cell damage was alleviated upon Parkin overexpression. Further, we demonstrated that SAL ameliorates mitochondrial damage, ROS release, and apoptosis in PC12 cells by enhancing Parkin-induced mitophagy. In vivo experiments confirmed that Parkin-mediated mitophagy activation alleviates OIPNP in rats. Together, our study found that Parkin-mediated mitophagy is involved in the pathogenesis of OIPNP and might be a potential therapeutic target for OIPNP.

## MATERIALS AND METHODS

### PC12 Cell Culture

Well-differentiated rat adrenal pheochromocytoma cell line (PC12) was purchased from the Cell Bank of the Chinese Academy of Sciences (25, 26). Cells were grown in RMPI-1640 supplemented with 10% fetal bovine serum (FBS, CLARK), streptomycin/penicillin (1%) at 37°C in a humidified incubator of 95% air and 5% CO_2_. The culture media was replaced every other day. When the cells reached confluency, the cells were subcultured or seeded into a 10 cm cell culture dish or multiwall plates per the experimental requirement.

### Liposome Transfection

The recombinant overexpression, knockdown, and control plasmids of Parkin without the fluorescent labels were constructed by Sangon Biotech (Shanghai) Co., Ltd. When the PC12 cells reached 60% to 70% confluency, cells were transfected with recombinant plasmids using Lipofectamine 2000, according to the manufacturer’s instructions. The transfection effect was tested at the protein level before drug stimulation.

### Western Blot Assay

The total protein of rat DRG tissue and PC12 cells was extracted using RIPA lysis buffer containing phenyl methane sulfonyl fluoride (PMSF) and phosphatase inhibitor. In addition, mitochondrial proteins were extracted from the same batch of samples using the Tissue or Cell Mitochondria Isolation Kit (C3601, Beyotime Biotechnology), according to the manufacturer’s instructions. The BCA Protein Assay Kit (P0010S, Beyotime Biotechnology) was used to determine the protein concentration of each sample. Sample proteins (40 μg) were separated by SDS-polyacrylamide gel electrophoresis and transferred to the PVDF membrane. The membranes were blocked for 1 h at room temperature with 5% non-fat milk and incubated overnight on a shaker at 4°C with primary antibodies against SQSTM1/p62 (#5114), Parkin (#2132), LC3B (#2775), Cleaved Caspase-3 (#9661) (1:1,000, Cell Signaling Technology, Beverly, MA) and Bcl-2 (12789-1-AP), Bax (50599-1-AP), β-actin (66009-1-Ig), VDAC1 (55259-1-AP) (1:1,000, Proteintech). After washing, the membranes were incubated with secondary antibodies (1:1,000) for 1 h at room temperature. Protein bands were detected using the MicroChemi chemiluminescence imaging system (DNR BioImaging Inc., Israel) with electrochemiluminescence (ECL). Finally, ImageJ was used to measure the gray value of protein bands quantitatively. β-actin and VDAC1-mito were used as internal controls for total and mitochondrial proteins, respectively. VDAC1-cyto represented VDAC1 in the cytoplasm.

### Immunofluorescence

Treated PC12 cells were washed thrice with PBS and fixed in 4% paraformaldehyde for 30 min, permeabilized with 0.1% Triton X-100 for 1 h, and blocked with 1% BSA for 1 h. DRG frozen sections were blocked with 5% BSA for 1.5 h. The samples were then probed at 4°C overnight with antibodies against Parkin, LC3II, and Tomm20, and incubated for 1 h with a secondary antibody at room temperature. Cell nuclei were stained for 10 min with DAPI. Finally, samples were washed and imaged with a fluorescence microscope (for cells ×600, for DRG ×400) (Zeiss LSM800, Germany).

### Mitotracker Red Staining

Mitotracker red CMXRos (C1035, Beyotime Biotechnology) was used to stain mitochondria in live cells (27). According to the manufacturer’s instructions, PC12 cells were incubated with 200 nM Mitotracker probes for 30 min at 37°C. After removing the working solution and adding it to the medium, PC12 cells were imaged with a fluorescence microscope (×200) (Olympus Inc., Tokyo, Japan). The fluorescence intensity was quantified using Image J (Bethesda, MD).

### Measurement of Mitochondrial ROS

Mitochondrial ROS was measured using MitoSOX Red Mitochondrial Superoxide Indicator (Invitrogen, M36008). PC12 cells were plated in 24-well plates in a complete culture medium. The treatment cells were then incubated with MitoSOX Red (5 μM) for 10 min at 37°C, washed with PBS thrice, and observed using a fluorescence microscope (×100) (Olympus Inc., Tokyo, Japan).

### Mitochondrial Membrane Potential

Mitochondrial membrane potential (ΔΨM) was assessed using the mitochondrial-specific fluorescent probe JC-1 (C2006, Beyotime Biotechnology) (28). JC-1 is an ideal probe for detecting ΔΨM. Polymerized JC-1 produces red fluorescence, while in a monomeric form, green fluorescence is produced. The ratio of red and green fluorescence reflects the change of ΔΨM. PC12 cells and purified mitochondria from DRG were incubated with JC-1 (5 μM) for 30 min at 37°C and observed by a fluorescence microscope (×200) (Olympus Inc., Tokyo, Japan) and fluorescence spectrophotometer, respectively (Bio-Rad Model 680).

### Reactive Oxygen Species Assay

PC12 cells were plated in 24-well plates in a complete culture medium. The treatment cells were then incubated with DCFH-DA (10 μM) for 20 min at 37°C, according to the protocol of the ROS assay kit (S00330, Beyotime Biotechnology). After washing the cells, three random microscopic fields were imaged per slide with a fluorescence microscope (×100) (Olympus Inc., Tokyo, Japan).

### Annexin V-FITC Cell Apoptosis Rate Assay

According to the instructions of the Annexin V-FITC apoptosis detection kit (C1062M, Beyotime Biotechnology), the corresponding reagents were added to 1 × 10^6^ PC12 cells, and the cells were incubated in the dark for 20 min before the ice bath (approximately 2 h). The apoptosis rate was detected and analyzed by flow cytometry (BD Biosciences).

### Animals

Two-month-old male Sprague–Dawley rats weighing 200–220 g were purchased from the Animal Center of Jilin University. All the animals were housed in 3 per cage, with free access to food and water ad libitum, maintaining a constant temperature of 23°C ± 1°C, relative humidity 55%±10%, and 12/12 h light/dark cycle during the experiments. The animals were acclimatized for 1 wk, and baseline measurements were taken before starting the experiment. Jilin University institutional animal ethics committee approved all animal studies (authorization number: 2019071606).

### Experimental Design and Animal Treatment

The rats were randomly divided into five groups of 10 animals each and assigned to normal control (NC), oxaliplatin control (OC), and three treatment groups. OC group and two treatment groups were injected with OXA (2.4 mg/kg dissolved in 5.0% glucose solution, Shanghai Hengrui Pharmaceutical Co., Ltd., China) intraperitoneally (ip) for 5 consecutive days, followed by 2 days of rest, for three cycles, as described previously (29). The total cumulative dose would then be 36 mg/m^2^, corresponding to >1,184 mg/m^2^ in humans simulating the clinical cumulative oxaliplatin dose causing OIPNP (5, 30). Then the two treatment groups were treated with SAL (dissolved in 5.0% glucose) at 50 (OC + S50) and 100 mg/kg, ip (OC + S100). NC rats received 1 mL 5% glucose ip (vehicle). The fifth group was normal rats receiving SAL alone at 100 mg/kg, ip (S100). Behavioral tests were conducted before and on *days 7*, *14*, and *21*, after first injection of drugs. DRGs were collected within 24 h after the final behavioral test.

### Mechanical Allodynia Testing

Rats were placed individually in a plastic enclosure on an elevated wire mesh floor and allowed to habituate for 30 min before testing. A series of 8 calibrated von Frey filaments (4, 6, 8, 10, 15, 26, 60, 100 g, Stoelting) were kept perpendicular to the center of the sole and avoided the foot pads. The filaments were maintained in an S-shape for no more than 8 s. The “up-and-down” method, originally described by Dixon (31) and Chaplan et al. (32), was used to measure and record a 50% mechanical withdrawal threshold (50% MWT). Briefly, testing was initiated with the 6 g filament. The next weaker filament was applied if a quick paw withdrawal was observed; otherwise, the next stronger filament was applied (33). The interval between 2 consecutive tests was 10 s. Each animal was measured thrice for the 50% MWT [50% MWT= (10Xf + κδ)/10,000, where Xf is the logarithmic value of the last filament used, κ is a tabular value for the pattern of six positive/negative responses, δ is the average logarithmic value between filaments], and the average value was taken as the final value (34). All measurements were done by one experimenter to avoid handling bias.

### Thermal Hyperalgesia

Thermal hyperalgesia of the paw was performed using a hot/cold plate (35) (Ugo Basile Biological Research Apparatus, Varese, Italy). The rat was put into the test room to adapt to the environment 1 h before the test. Animals were then placed into an acrylic box (22.5 × 22.5 cm^2^) on a cold plate as the floor. The temperature of the cold plate was kept constant at 4 ± 1°C (36), and the hot plate at 52 ± 0.2°C (37). The latency of the first paw flicking or paw licking was considered the pain threshold (38, 39). The cut-off time was set at 60 s to avoid frostbiting the paws, whereas the cut-off time to avoid scalding was set at 15 s. The cut-off times were calculated from the time the animal’s paw was kept on the plate. The measurement was repeated thrice for each rat, with an interval of 10 min between each measurement.

### Cold Chemical Allodynia

With the animals atop the wire mesh frame, 0.05 mL of acetone was sprayed on the ventral center of the hind paw with a micro syringe and the timer was started. If the rat did not respond within 20 s, it was recorded as 0. However, if the rat responded to the cooling effect of acetone within the first 20 s, then the rat’s response was evaluated for another 20 s (40, 41). The response was graded on a 4-point scale: 0: no response; 1: quick withdrawal or flicking the paw; 2: prolonged withdrawal or repeated flicking; and 3: repeated flicking with paw licking (39). Each paw was sprayed with acetone three times alternately, with an interval of 5 min. Cumulative scores were then generated by adding the six scores for each rat; the minimum score was 0, and the maximum was 18.

### Transmission Electron Microscope

Rats in each group were perfused with a mixture of paraformaldehyde and glutaraldehyde solution at a final concentration of 4%. Subsequently, the DRG at L4-L6 was removed and fixed overnight with a pre-cooled 4% glutaraldehyde solution. Samples were washed with distilled water and fixed in 1% osmic acid for another 1 h. Finally, the samples were dehydrated with 50%, 70%, 80%, 90%, and 100% ethanol for 15 min each and embedded in epoxy resin. Semi-thin sections (300 nm) were stained with 0.5% toluidine blue and examined for localization under an optical microscope. Ultrathin sections (65 nm) were stained with lead citrate and 3% uranyl acetate and examined for mitochondrial morphology using a transmission electron microscope (TEM) (HITACHI h-7500, Japan).

### Measurement of Plantar Intraepidermal Nerve Fiber Density

Plantar skin (1 cm × 1 cm) was fixed with 4% paraformaldehyde for 48 h. Section samples (5 μm) were rehydrated and incubated with PGP 9.5 antibody (ab101986, 1:250, Abcam, UK). The sections were washed and stained with hematoxylin, and the brown profile indicated positive. Images were acquired at ×400 magnification, nerve fibers crossing the epidermal junction (PGP 9.5 immunoreactive profiles) were quantified (42), and IENF/mm was calculated (43).

### Nissl Staining

Nissl staining droplets were placed on paraffin section samples and washed with clean water. The Nissl bodies turned dark blue with 0.1% glacial acetic acid, and the excess water was removed and observed under a light microscope (×600) (Olympus Inc., Tokyo, Japan).

### TUNEL Assay

The terminal deoxynucleotidyl transferase (TdT) dUTP nick-end labeling (TUNEL) assay detected apoptosis in DRG. Frozen sections were fixed in 4% paraformaldehyde for 1 h, then incubated with 0.5% Triton X-100 for 5 min at room temperature and washed thrice with PBS between each step. Staining was performed according to the TUNEL assay kit (C1089, Beyotime Biotechnology) and DAPI instructions. Finally, three random fields of each slide were imaged with a fluorescence microscope (×400) (Olympus Inc., Tokyo, Japan), and the positive rate was calculated.

### Enzyme-Linked Immunosorbent Assay for ROS in DRG

L4-L5 fresh DRG (500 mg) was extracted for homogenization. The supernatant was extracted and tested for ROS using the enzyme-linked immunosorbent assay (ELISA) ROS kit (Enzyme-linked Biotechnology Co., Ltd, Shanghai, China), according to the manufacturer’s instructions. The absorbance of each sample was measured with a 450 mm spectrophotometer, and the actual ROS concentration was calculated.

### Statistical Analysis

The results were presented as means ± SD. Statistical analyses were performed using GraphPad Prism 8.0 (GraphPad Software). Data were analyzed by one-way analysis of variance (ANOVA) followed by Tukey’s test for comparison between control and treatment groups. The Kruskal–Wallis test analyzed nonparametric data. Welch’s ANOVA test was used when the variance was not uniform (Brown–Forsythe test). *P* < 0.05 was considered significant.

## RESULTS

### OXA Reduces Parkin-Mediated Mitophagy and ROS Release in DRG of Neuropathic Rats

To explore how OXA affects Parkin-mediated mitophagy and ROS levels in rat DRG, we extracted DRG from the oxaliplatin control (OC) group on *days 0*, *7*, *14*, and *21*. Western blot (WB) and tissue immunofluorescence results showed that, with the increase in time, OXA reduced the expression of Parkin, and the expression was the lowest on the 21st day (β3-Tublin is a neuron-specific protein).

**Figure 1.**
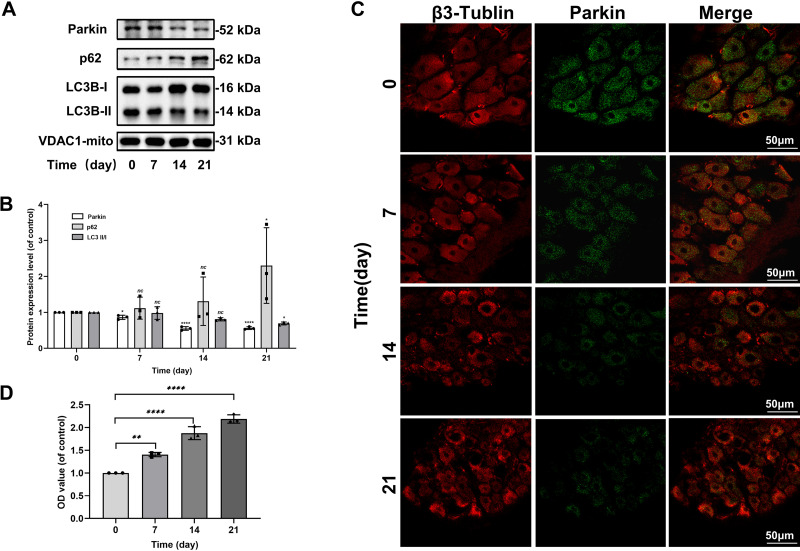
OXA reduces Parkin-mediated mitophagy and enhances ROS release in DRG of neuropathic rats. *A*: the expression of Parkin, p62, and LC3B in the DRG of oxaliplatin-induced pain rats at each time point was analyzed using Western blots. *B*: quantification of Parkin, p62, and LC3 II/I immunoblots. *C*: representative image of immunofluorescence double staining of Parkin and β3-Tubulin in DRG of oxaliplatin-induced pain rats at each time point (×400). *D*: the OD value of ROS in DRG of oxaliplatin-induced pain rats was quantified. Data represent the means ± SE of multiple independent experiments, each done in triplicate. Significant differences between groups are indicated as ****P < 0.0001, **P < 0.01, *P < 0.05. DRG, dorsal root ganglion; OD, optical density; OXA, oxaliplatin; ROS, reactive oxygen species.

LC3-I stands for autophagy precursor, LC3-II stands for mature autophagosome, and LC3 II/I represents the degree of conversion of autophagy precursor to mature autophagosome (44). An adaptor protein, p62, regulates the movement of LC3 toward the damaged mitochondria in ubiquitin-mediated mitophagy (45). We found a gradual decrease in LC3 II/I, but the expression of p62 increased over time (Fig. 1, *A–C*). OXA reduced mitophagy flux in DRG of pain model and inhibited Parkin-mediated mitophagy. Similarly, ELISA also showed a gradual increase in the release of ROS in DRG (Fig. 1*D*).

### OXA Inhibits Parkin-Mediated Mitophagy, Leading to Mitochondrial Damage and Apoptosis in PC12 Cells

**Figure 2.**
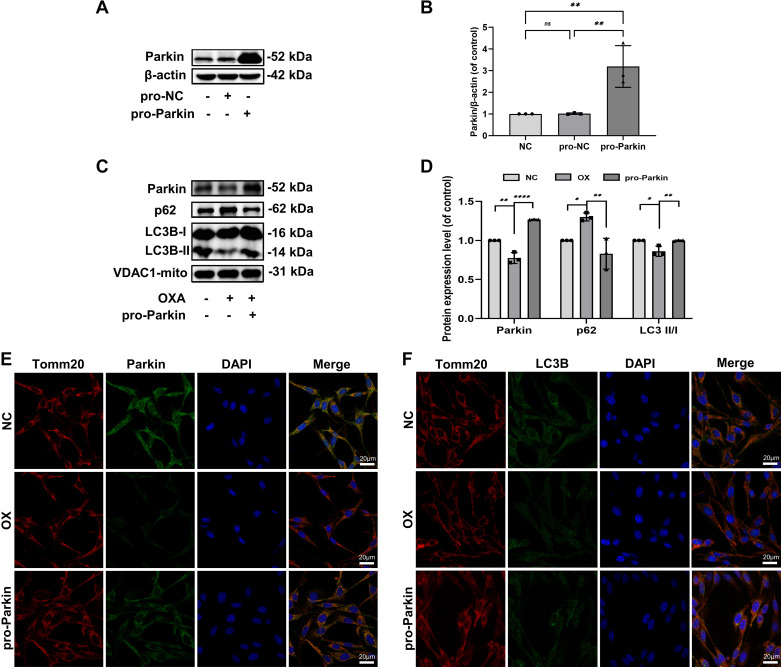
Changes in mitophagy upon Parkin overexpression in OXA stimulated PC12 cells. *A*: Western blots show the expression of Parkin. *B*: quantification of Parkin immunoblots. *C*: Western blot analysis demonstrating the expression of Parkin, p62, and LC3B. *D*: quantification of Parkin, p62, and LC3 II/I immunoblots. *E*: representative image of immunofluorescence double staining of Parkin and Tomm20 in PC12 cells (×600). *F*: representative image of immunofluorescence double staining of LC3B and Tomm20 in PC12 cells (×600). Data represent the means ± SE of multiple independent experiments, each done in triplicate. Significant differences between groups are indicated as ****P < 0.0001, **P < 0.01, * *P*< 0.05. NC, normal control; OX, OXA, oxaliplatin.

To further verify that OXA inhibits Parkin-mediated mitophagy and to confirm whether the reduced expression of Parkin was correlated with mitochondrial damage and increased apoptosis, we overexpressed Parkin in PC12 cells and then examined mitophagy, mitochondrial function, and apoptosis. The WB results showed an increased expression of Parkin intracellularly and on the mitochondria (Fig. 2, *A*, *B*, *E*). Moreover, OXA stimulation in PC12 cells significantly decreased mitochondrial Parkin and LC3 II/I while increasing p62 levels, indicating that the mitochondrial autophagic flux was impaired. In the pro-Parkin group (PC12 cells were first overexpressed with Parkin and then stimulated with 3 μM OXA (46)), LC3 II/I was increased, and p62 was decreased compared with the OX group (PC12 cells were stimulated with only 3 μM OXA), and the autophagic flux was restored (Fig. 2, *C*, *D*).

To better understand mitophagy, Tomm20 (a mitochondrial membrane marker protein) and LC3B (a recognized autophagy marker protein) were used to stain PC12 cells stimulated by OXA. Fluorescence staining confirmed the reduction in Parkin-mediated mitophagy. However, Parkin overexpression enhanced the fluorescence intensity of double staining (Fig. 2, *E*, *F*).

**Figure 3.**
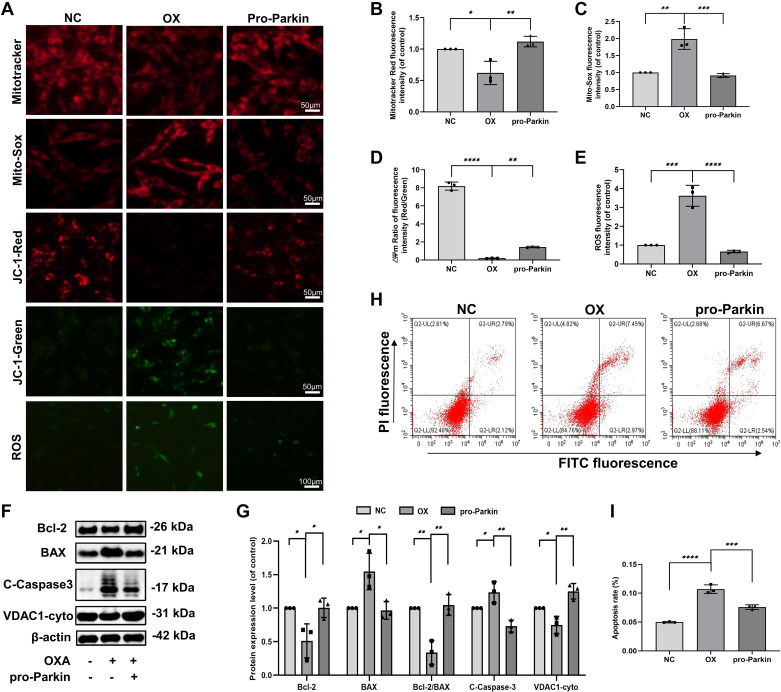
The overexpression of Parkin is closely associated with mitochondrial dysfunction and apoptosis in OXA-stimulated PC12 cells. *A*: Mitotracker was used to stain mitochondria, Mito-Sox was used to measure the mitochondrial ROS, JC-1 was used to measure the mitochondrial membrane potential (△Ψm), and ROS Assay Kit was used to detect intracellular ROS in PC12 cells. *B–E*: the fluorescence intensity of Mitotracker, Mito-Sox, △Ψm (Red/Green) and ROS was quantified. *F*: Western blots analyze the expression of Bcl-2, BAX, C-Caspase3, and VDAC1-cyto. *G*: quantification of Bcl-2, BAX, Bcl-2/BAX, C-Caspase3, and VDAC1-cyto immunoblots. *H*, *I*: annexin V-FITC flow cytometry and statistical analysis of apoptosis rate. Data represent the means ± SE of multiple independent experiments, each done in triplicate. Significant differences between groups are indicated as ****P < 0.0001, ***P < 0.001, **P < 0.01, *P < 0.05. NC, normal control; OX, OXA, oxaliplatin; ROS, reactive oxygen species.

We found that OXA significantly reduced the expression of VDAC1-cyto and the fluorescence intensity of Mitotracker in PC12 cells (Fig. 3, *A*, *F*). The results showed a reduction in the number of live mitochondria. OXA also decreased mitochondrial membrane potential (△Ψm) and increased the fluorescence intensity of MitoSOX and intracellular ROS in PC12 cells. However, overexpression of Parkin reversed OXA-induced mitochondrial number reduction and function damage in PC12 cells (Fig. 3, *A–E*).

Mitochondrial damage mediates apoptosis. The WB showed that the ratio of Bcl-2 (anti-apoptotic index) to Bax (pro-apoptotic index) was significantly reduced, and Cleaved-Caspase3 (C-Caspase3) was significantly increased in PC12 cells with OXA stimulation compared with the untreated controls (Fig. 3, *F*, *G*). Flow cytometry results further indicated that OXA significantly increased apoptotic and necrotic cells. However, apoptosis was reduced upon Parkin overexpression (Fig. 3, *H*, *I*). Taken together, these results suggest that Parkin-mediated reduction of mitophagy might be necessary for OXA-induced mitochondrial damage and apoptosis.

### SAL Enhances Parkin-Mediated Mitophagy, Dampens Mitochondrial Damage, and Reduces Apoptosis in PC12 Cells

**Figure 4.**
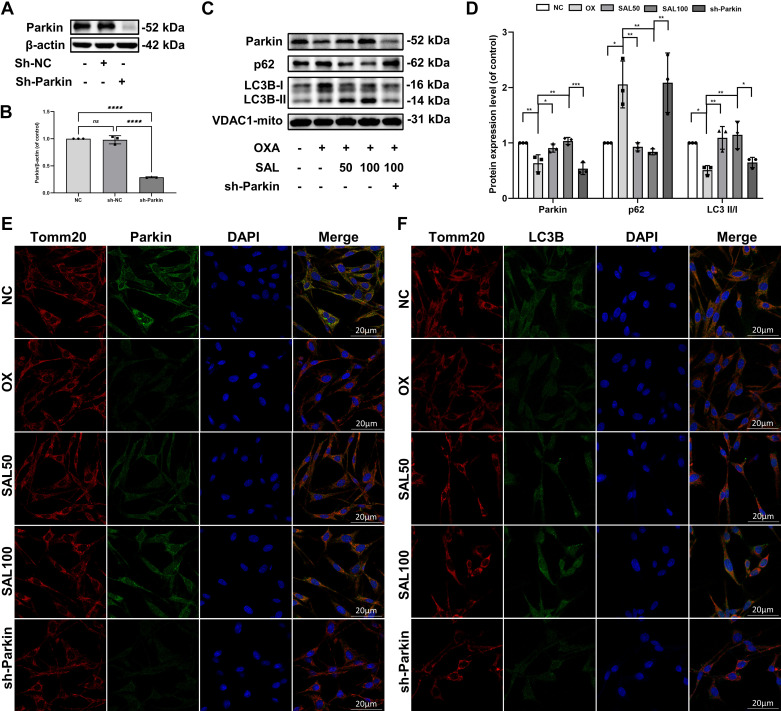
Changes in mitophagy upon Parkin inhibition in OXA and SAL stimulated PC12 cells. *A*: the expression of Parkin was evaluated by Western blots. *B*: quantification of Parkin immunoblots. *C*: the expression of Parkin, p62 and LC3B was evaluated by Western blots. *D*: quantification of Parkin, p62, and LC3 II/I immunoblots. *E*: representative image of immunofluorescence double staining of Parkin and Tomm20 in PC12 cells (×600). *F*: representative image of immunofluorescence double staining of Parkin and Tomm20 in PC12 cells (×600). Data represent the means ± SE of multiple independent experiments, each done in triplicate. Significant differences between groups are indicated as ****P < 0.0001, **P < 0.01, *P < 0.05. NC, normal control; OX, OXA, oxaliplatin; SAL, salidroside.

It has been reported that SAL can enhance mitophagy in nucleus pulposus cells to reduce cell damage. We wanted to examine whether SAL affects mitochondrial function and apoptosis by regulating Parkin-mediated mitophagy in PC12 cells. Therefore, we transfected PC12 cells with liposomes to downregulate Parkin (sh-P) and treated them with two concentrations of SAL. The results showed a reduction in the expression of Parkin (Fig. 4, *A*, *B*).

WB showed that SAL increased LC3 II/I, decreased p62, and increased double staining fluorescence intensity of Tomm20 and LC3B in a dose-dependent manner, indicating that mitophagy was activated and autophagic flux was restored. Upon Parkin knockdown, LC3-I and LC3-II were reduced in the sh-Parkin group compared with the SAL100 group, indicating that both autophagy precursors and mature autophagic vesicles were reduced. At the same time, the expression of p62 in the sh-Parkin group was significantly increased, suggesting that p62 accumulation and mitochondrial autophagic flux were impaired. Immunofluorescence results demonstrated a reduced LC3B on mitochondria when Parkin was downregulated (Fig. 4, *C*–*F*).

**Figure 5.**
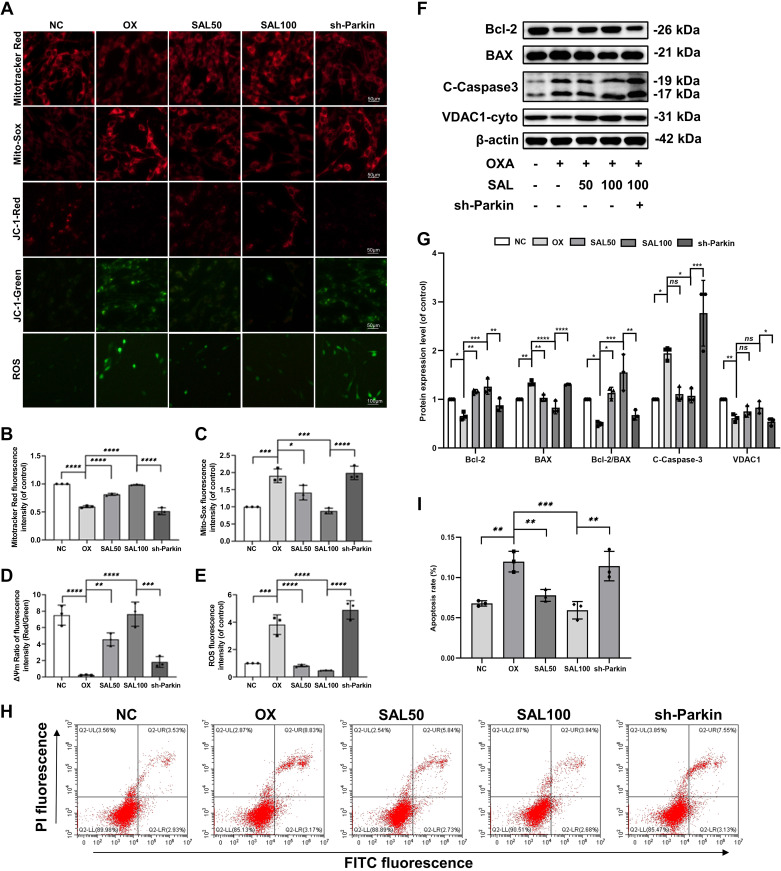
The therapeutic effect of SAL on OXA-stimulated mitochondrial dysfunction and apoptosis in PC12 cells upon Parkin inhibition. *A*: Mitotracker was used to stain mitochondria, Mito-Sox was used to measure the mitochondria ROS, JC-1 was used to measure the mitochondrial membrane potential (△Ψm), and ROS Assay Kit was used to detect intracellular ROS in PC12 cells. *B*–*E*: the fluorescence intensity of Mitotracker, Mito-Sox, △Ψm (Red/Green) and ROS was quantified. *F*: Bcl-2, BAX, C-Caspase3, and VDAC1-cyto were evaluated using Western blots. *G*: quantification of Bcl-2, BAX, Bcl-2/BAX, C-Caspase3, and VDAC1-cyto immunoblots. *H*, *I*: annexin V-FITC flow cytometry and statistical results of apoptosis rate. Data represent the means ± SE of multiple independent experiments, each done in triplicate. Significant differences between groups are indicated as ****P < 0.0001, ***P < 0.001, **P < 0.01, *P < 0.05. NC, normal control; OX, OXA, oxaliplatin; SAL, salidroside.

With the enhancement of mitophagy, the expression of VDAC1-cyto, the fluorescence intensity of Mitotracker and the △Ψm increased, while the fluorescence intensity of MitoSOX and ROS levels decreased in PC12 cells. Knockdown of Parkin prevented the therapeutic effect of SAL on the mitochondria of PC12 cells. The number of mitochondria decreased, △Ψm decreased, and ROS production by mitochondria and cells increased (Fig. 5, *A–E*).

We found that in PC12 cells treated with SAL, Bcl-2/Bax was significantly increased, and C-Caspase3 and the apoptosis rate were significantly decreased compared with the OX group in a dose-dependent manner. Knockdown of Parkin in PC12 cells reversed the anti-apoptotic effect of SAL; Bcl-2/Bax was significantly decreased, and C-Caspase3 and apoptosis rates were significantly increased (Fig. 5, *F–I*). Together, our results indicate that SAL could enhance Parkin-mediated mitophagy to eliminate damaged mitochondria and increase cell survival in PC12 cells.

### SAL Alleviates Oxaliplatin-Induced Neuropathy and Pain in Rats

**Figure 6.**
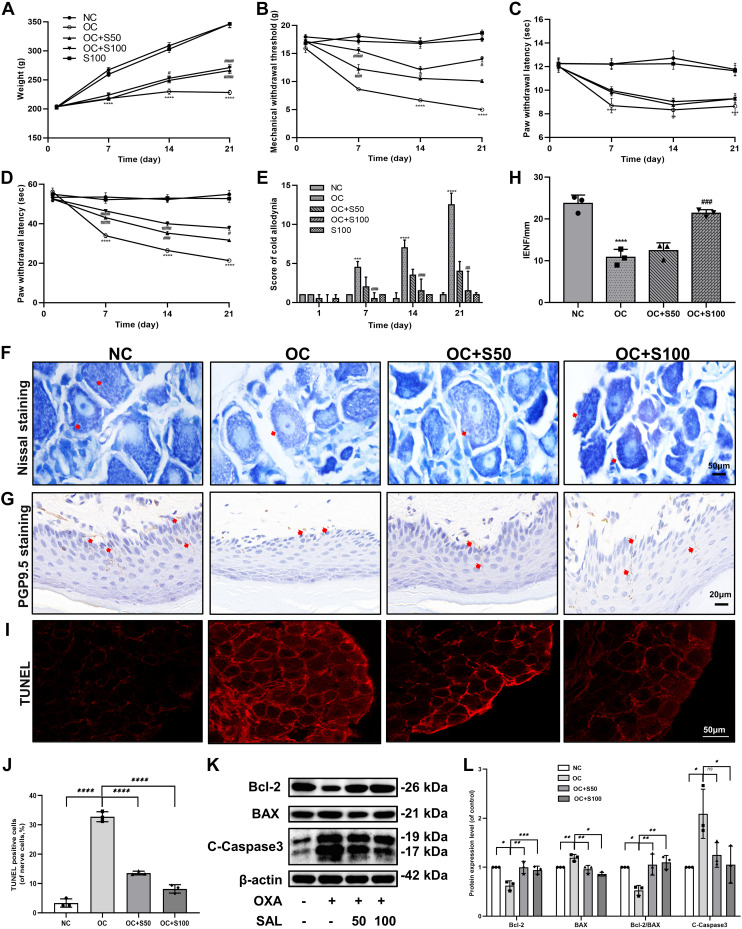
Effect of SAL on behavioral changes and DRG in oxaliplatin-induced pain rats. *A*: body weights. *B*: mechanical allodynia. *C*: hot (52 ± 0.2 °C) hyperalgesia. *D*: cold (4 ± 1°C) hyperalgesia. *E*: acetone cold chemical allodynia. *F*: Nissl body staining of the DRG. *G*: detection of intraepidermal nerve fiber (IENF) density in rat plantar epidermis. *H*: quantification of IENFD. *I*: TUNEL assay was used to evaluate the apoptosis in DRG in the different groups. *J*: positive apoptotic cells were quantified. *K*: quantification of Bcl-2, BAX, and C-Caspase3 immunoblots. *L*: quantification of Bcl-2, BAX, Bcl-2/BAX, and C-Caspase3 immunoblots. Results were expressed as means ± SE (*n* = 10). Intergroup comparison: ****P < 0.0001, ***P < 0.001, **P < 0.01, *P < 0.05 vs. NC, *####P* < 0.0001, *###P* < 0.001, *##P* < 0.01, *#P* < 0.05 vs. OC. NC, normal control; OC, oxaliplatin control; OXA, oxaliplatin; SAL, salidroside.

A model of OIPNP was established by continuous intraperitoneal injection of small OXA doses and then treated with SAL. Pain behavior was evaluated by 50% mechanical withdrawal threshold (50% MWT), hot and cold pain threshold, and acetone cold hyperalgesia score. Body weight was measured once a week to observe the systemic toxicity of OXA. Although no animal died during the study, OXA-injected animals were weaker and less active. The OC and the two treatment groups showed slow weight gain compared with the NC group. The OC group even showed weight loss in the third week (Fig. 6*A*). After OXA treatment, the 50% MWT and cold and hot paw withdrawal threshold were lower than those in the NC group, accompanied by an increase in acetone cold hyperalgesia score, suggesting that the neuropathic pain model was successfully induced. On the other hand, SAL increased MWT by 50%, cold paw withdrawal threshold, and acetone cold hyperalgesia score in a dose-dependent manner. However, it did not affect the hot paw withdrawal threshold. Moreover, SAL treatment alone did not affect either animal weight or behavioral changes compared with the NC group (Fig. 6, *B–E*).

DRG Nissl staining can be used as a sensitive marker to determine whether nerve cells are damaged and show the pathological morphology of nerve cells (47). After OXA treatment, the staining of Nissl bodies in the DRG of rats was light and sparse, and the nuclei were skewed or binucleated. SAL increased the Nissl body staining, but the skewness of the nuclei and the binucleation persisted (Fig. 6*F*).

The measurement of plantar intraepidermal nerve fiber density (IENFD) can be used in the early diagnosis of peripheral neuropathy (48). The nerve fiber density in the foot epidermis decreased after OXA treatment, whereas SAL prevented the OXA-induced loss of nerve fibers in the epidermis (Fig. 6, *G, H*).

The apoptosis of cells in DRG was estimated by TUNEL assay and apoptosis-related protein expression (Fig. 6, *I, J*). We observed that the apoptosis of DRG cells in the OC group was significantly higher than in the control group. However, treatment with SAL reversed this pathology in vivo (Fig. 6, *K, L*). Therefore, our results suggest that SAL alleviates neuropathy and pain in OXA-induced neuropathic rats.

### SAL Enhances Parkin-Mediated Mitophagy and Attenuates Mitochondrial Damage in Oxaliplatin-Induced Neuropathy in Rat DRG

In the above experiments, we have found that SAL can reverse the damage caused by OXA in PC12 cells by enhancing Parkin-mediated mitophagy. We further verified the mechanism by which SAL alleviates DRG injury in vivo.

**Figure 7.**
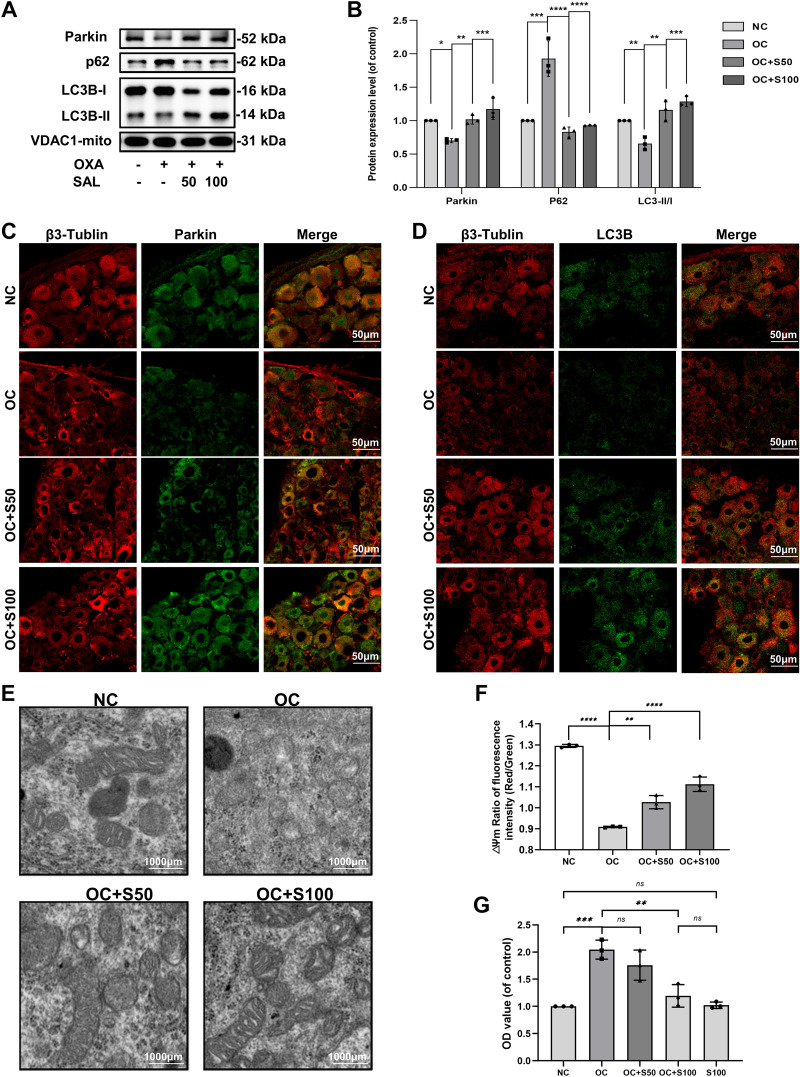
Salidroside (SAL) enhances Parkin-mediated mitophagy and reduces ROS release in the DRG of neuropathic rats. *A*: the expression of Parkin, p62, and LC3B was evaluated by Western blots. *B*: quantification of Parkin, p62, and LC3 II/I immunoblots. *C*: representative image of immunofluorescence double staining of Parkin and β3-Tubulin in the DRG (×400). *D*: representative image of immunofluorescence double staining of LC3B and β3-Tubulin in the DRG (×400). *E*: representative image of mitochondrial morphology in the DRG (×8,000). *F*: fluorescence intensity quantification of △Ψm (Red/Green) in the DRG. *G*: the OD value of ROS in the DRG of pain rats. Data represent the means ± SE of multiple independent experiments, each done in triplicate. Significant differences between groups are indicated as ***P < 0.001, **P < 0.01, *P < 0.05. DRG, dorsal root ganglion; NC, normal control; OC, oxaliplatin control; OD, optical density; ROS, reactive oxygen species.

WB results showed that the expression of Parkin in the OC + S50 group and OC + S100 group increased compared with the OC group. Furthermore, the expression of LC3-I decreased, and LC3-II increased, increasing LC3 II/I, the expression of p62 also decreased (Fig. 7, *A, B*). These results indicate that SAL increases mitophagy flux in DRG.

The DRG neurons of the NC group were complete in shape, with uniform β3-Tubulin staining, while the DRG neurons of other groups were shrunken and had an uneven β3-Tubulin staining (Fig. 7*C*). However, the Parkin and β3-Tubulin double staining fluorescence intensity of OC + S50 group and OC + S100 group were higher than that of OC group. Compared with the OC group, the fluorescence intensity of Tomm20, LC3B, and their double staining increased in the SAL treatment group (Fig. 7*D*).

Transmission electron microscopy (TEM) results showed that the structure of the mitochondrial bilayer membrane in the DRG neurons of rats in the NC group was clear, and the mitochondrial cristae were orderly and dense. On the contrary, the mitochondria of the OC group were swollen and vacuolar, and the bilayer membrane and cristae structure were unclear. Mitochondrial morphology returned to normal in the treatment group (Fig. 7*E*).

In the OC group, △Ψm of the DRG mitochondria decreased significantly, and the mitochondrial membrane was damaged. △Ψm increased after SAL treatment in a dose-dependent manner (Fig. 7*F*). Moreover, the amount of ROS in the DRG was significantly reduced in the SAL treatment group (Fig. 7*G*). These results indicated that SAL reversed OXA-induced reduction of Parkin-mediated mitophagy and alleviated mitochondrial damage and release of ROS in the DRG.

## DISCUSSION

Oxaliplatin exhibits both central and peripheral neurotoxicity, which can manifest after initial treatment and persist for months or even years, with severity dependent on cumulative dosage (49, 50). This study aims to elucidate the underlying mechanism of chronic peripheral nerve damage and pain induced by OXA. Peripheral neurons suffering from chemotherapeutic drug injury can produce ROS, and ROS can also lead to mitochondrial damage. The damaged mitochondria, in turn, can generate a large amount of ROS as positive feedback, forming a vicious circle. Many in vivo and in vitro studies verified the mechanism of ROS-induced peripheral nerve pain. ROS can directly increase TRPA1 and TRPV1 receptors when examined in OXA-treated DRGs cultured in vitro (51). ROS can induce the release of IL-1β by increasing NLRP3 inflammasome and induce peripheral nerve pain in mice (52). It has also been found that ROS produced by OXA stimulation can directly cause the release of TNF-α and IL-6 inflammatory factors and caspase-3-mediated apoptosis, leading to peripheral nerve pain (53). ROS can also sensitize central spinal neurons to pain (54). Therefore, ROS-induced oxidative stress is an essential part of peripheral nerve pain. This study demonstrates that OXA induces ROS levels in DRG, corroborating findings from previous investigations.

Parkin-mediated mitophagy is crucial to maintaining cell homeostasis and a dynamic mitochondrial network. Autophagosomes selectively engulf damaged or dysfunctional mitochondria to reduce ROS release, essential for highly differentiated and refractory peripheral neurons. Our results suggest that OXA affects Parkin-mediated mitophagy, and increased ROS contributes to peripheral nerve injury and pain. Enhanced mitochondrial autophagy, however, mitigates OXA-induced cellular and DRG damage and attenuates peripheral nerve pain, thereby highlighting the significance of targeting mitophagy for the effective management of OIPNP.

Mitophagy is a type of selective autophagy which is highly regulated. It can be further divided into receptor-mediated and ubiquitin-mediated mitophagy. PINK1-Parkin mediates the classical pathway of ubiquitin-mediated mitophagy (18). Under physiological conditions, PINK1 is imported into mitochondria through the translocase of the outer membrane (TOM) and translocase of the inner membrane (TIM) complexes. It is cleaved into fragments by mitochondrial processing peptidase (MPP) and presenilin-associated rhomboid-like protein (PARL) in the inner membrane (55–58). The fragments are then transferred outside the mitochondria and hydrolyzed by the proteasome (59–62). In contrast, Parkin is an E3 Ubiquitin ligase in the cytoplasm with an N-terminus ubiquitin-like domain (UblD) (63). UblD inhibits Parkin activity under physiological conditions (64, 65).

When OXA reduces the mitochondrial membrane potential, Parkin’s E3 ubiquitin ligase activity is activated and translocates to the damaged mitochondrial outer membrane (66–69). Parkin induces more poly-Ub on mitochondria (typical mitochondrial poly-Ub includes Lys48 and Lys63, whereas atypical poly-Ub includes Lys11 and Lys6) (51, 52, 63, 70). p62 recognizes Poly-Ub and induces the movement of LC3 towards the damaged mitochondria. Our results showed that the gradual decrease of LC3 II/I was accompanied by a gradual increase of p62, indicating that the transition from autophagy precursor to mature autophagosome was reduced, and mitochondrial autophagic flux was impaired. The increased accumulation of p62 on mitochondria might result from reduced mature autophagosomes and reduced transport of the adapter p62 to the injured mitochondria. Eventually, autophagosomes phagocytose damaged mitochondria, and lysosomes further digest autophagosomes. In vitro experiments demonstrated that Parkin overexpression attenuates the inhibitory effect of OXA on mitophagy in PC12 cells, leading to increased cell survival rate and alleviated apoptosis. Thus, Parkin-mediated mitophagy serves as a pathogenic mechanism underlying OIPNP, and p62 serves both as an autophagosome transporter and a response to autophagic flux changes (44).

The drugs for the treatment of CIPNP should meet the following requirements: they can relieve hyperalgesia and allodynia, can treat peripheral nerve injury, have no side effects on the body, and do not promote tumor growth. There is no clinically recognized specific drug for OIPNP. We have found that SAL can reverse OXA-induced mitochondrial damage, ROS release, and apoptosis by enhancing Parkin-mediated mitophagy in PC12 cells. We, therefore, speculated whether SAL could target Parkin in the DRG to relieve peripheral nerve injury and pain caused by OXA. We set up low-dose (50 mg/kg) and high-dose (100 mg/kg) SAL treatment groups according to the experimental dosage of SAL reported in the literature and carried out the experiment in OIPNP rat models (53).

SAL alleviated OXA-induced mechanical and cold pain in a dose-dependent manner, but it did not relieve heat pain. Although the treatment effect of SAL did not restore the pain behavior to the pre-model, the pain hypersensitivity and allodynia were alleviated at all time points in the SAL-treated group compared with the OXA group. However, the reason why SAL did not relieve heat pain may be related to OXA-activated receptors, and SAL might reduce the expression of TRPA1 but not TRPV1 in neurons. It is also possible that individual differences in rats are large and statistical differences could occur due to smaller sample sizes. Therefore, further experiments are needed to explore the effect of SAL on peripheral nerve heat pain in rats.

Intraperitoneal injection of SAL (100 mg/kg) alone did not change the body weight of rats, various pain thresholds, pain behavior, or the production of ROS in the DRG. Intraperitoneal injection of SAL, in combination with OXA, had a therapeutic effect. However, the toxic effects of SAL should be further verified in other types of gastrointestinal tumor cells and tumor-bearing rats.

We also demonstrated that SAL attenuated peripheral nerve injury induced by OXA. Since peripheral nerves include neuronal cell bodies, synapses, and nerve endings, we detected the neuronal cell bodies using Nissl bodies and the neuronal endings using IENFD. In addition, nerve conduction velocity detection should be performed to evaluate the damage to neural synapses.

For this study on the mechanism of OPINP, only male SD rats were utilized in vivo. The focus of this experiment was not on gender differences; however, it is important to note that sex differences do exist in clinical practice regarding peripheral nerve pain induced by chemotherapy drugs. Furthermore, different chemotherapy drugs exhibit varying gender differences and mechanisms of action (54, 71). In some animal experiments, there was no significant difference in OIPNP between female and male rats; however, the therapeutic response varied, potentially attributed to the secretion of the endocannabinoid system and sex hormones, alteration changes in spinal microglia, and changes in central astrocyte receptors (72–74). Nevertheless, further investigation is required to explore the variations and treatment disparities concerning Parkin-mediated mitophagy in both genders.

Finally, to validate the therapeutic mechanism of SAL in vivo, we isolated mitochondria from rat DRG neurons, examined the mitochondrial morphology and function, and the Parkin-mediated mitophagy pathway at the molecular level. The experimental results showed that SAL enhanced Parkin-mediated mitophagy in rat DRG neurons, reduced damaged mitochondria, and restored mitochondrial function, thereby reducing oxidative stress and apoptosis in peripheral neurons. Therefore, SAL may alleviate OXA-induced peripheral nerve pain by enhancing Parkin-mediated mitophagy and the targeting of Parkin-mediated mitophagy represents management of OIPNP.

## DATA AVAILABILITY

Data will be made available upon reasonable request.

## GRANTS

This research was funded by Jilin Province Science and Technology Development Plan Project and Natural Science Foundation of Jilin Province, Grant Numbers are 3D5206062430 and 3D5230920430, respectively.

## DISCLOSURES

No conflicts of interest, financial or otherwise, are declared by the authors.

## AUTHOR CONTRIBUTIONS

T.Z. conceived and designed research; T.Z., J.L., and C.Z. performed experiments; L.L., P.C., C.Z., and C.C. analyzed data; G.Z., L.L., and P.C. interpreted results of experiments; J.L. prepared figures; T.Z. drafted manuscript; G.Z. and K.L. edited and revised manuscript; G.Z. approved final version of manuscript.
